# Feeding Crude Glycerin to Finishing Iberian Crossbred Pigs: Effects on Growth Performance, Nutrient Digestibility, and Blood Parameters

**DOI:** 10.3390/ani11082181

**Published:** 2021-07-23

**Authors:** Silvia Martínez-Miró, Josefa Madrid, Miguel José López, Juan Orengo, Cristian Jesús Sánchez, Fuensanta Hernández

**Affiliations:** Department of Animal Production, Faculty of Veterinary Science, Regional Campus of International Excellence “Mare Nostrum”, University of Murcia, 30100 Murcia, Spain; alimen@um.es (J.M.); mjlopeza@um.es (M.J.L.); jorengo@um.es (J.O.); cristianjesus.sanchez@um.es (C.J.S.); nutri@um.es (F.H.)

**Keywords:** crude glycerin, Iberian × Duroc, growth performance, digestibility, blood parameters

## Abstract

**Simple Summary:**

Crude glycerin can be used as a substitute for some feed ingredients in white pig diets. However, there are few reports on its use in Iberian crossbred pigs. This study aimed to evaluate the effects of three levels of crude glycerin (0, 50, and 100 g/kg) on performance parameters, nutrient digestibility, and blood parameters of Iberian × Duroc pigs during the last phase of fattening. The results show that the addition of glycerin did not affect average daily gain, average feed intake, or feed conversion ratio. Digestibility of dry matter, organic matter, and crude protein and serum parameter concentrations were statistically similar among the evaluated treatments. It is concluded that crude glycerin up to 100 g/kg could be included in the diets of Iberian pigs for the last fattening phase with no negative effects.

**Abstract:**

A total of 192 Iberian × Duroc pigs kept under intensive conditions were used to investigate the effects of feeding crude glycerin on growth performance, nutrient digestibility, and blood parameters. Animals were blocked by initial body weight (96.0 ± 11.3 kg) and allotted to pens (16 pigs per pen). Pens were assigned randomly to one of three dietary treatments (four pens per treatment). Dietary treatments contained 0, 5, or 10% of crude glycerin proportionally substituting for wheat (G0, G5, and G10, respectively). Diets were formulated to be isoenergetic and isoaminoacidic. No significant effect of crude glycerin was observed on average daily gain, average feed intake, or feed conversion ratio. The apparent total tract digestibility of dry matter, organic matter, and crude protein was no different between treatments. Total serum protein, albumin, glucose, insulin, and IGF-1 were not affected by glycerin inclusion. In conclusion, crude glycerin up to 100 g/kg can be included in the diets of finishing Iberian crossbred pigs without any negative effect on growth performance, digestibility, or serum metabolic parameters.

## 1. Introduction

The increasing demand for transportation fuels, coupled with decreasing crude oil reserves and a growing awareness of climate change has increased global interest in biodiesel. Biodiesel is produced by transesterification of vegetable oils or animal fats with an alcohol present as a catalyst. This reaction yields a considerable amount of crude glycerin as a byproduct, approximately 10% by weight of the biodiesel produced [1]. During recent years, there has been much interest in using crude glycerin as a feed ingredient for livestock. Several studies have reported that crude glycerin is well digested and utilized by pigs as a source of energy [2,3,4,5]. Different concentrations of crude glycerin have shown no effects on growth performance, carcass composition, or meat quality: up to 10% [5,6,7,8,9], 14% [10], and 16% [11]. Most studies conducted on pigs have focused on the use of crude glycerin to feed commercial and conventional breeds such as Large White and Landrace, with a slaughter weight of about 110 kg, but very little information is available regarding its use as a feed ingredient in diets for traditional pigs with high slaughter weight such as Iberian pigs (IB).

The IB is an autochthonous breed in the southwest of Spain for which the traditional production system is in outdoor conditions, based on the rearing of pure Iberian pigs [12]. The productivity of the sows (fewer than 14 weaned piglets /sow/year) and the fattening pigs is low (feed conversion ratio from 25 to 160 kg body weight (BW) greater than 4.0) [12,13]. However, the marbling of Iberian pig meat is typically abundant and evident, much more intense than the meat from commercial genotypes, which is a direct consequence of the high intramuscular fat (IMF) content [14]. The IB sector is of great economic importance in Spain due to the high acceptance of premium cured products. The increasing production of pigs of the IB type has allowed the sector to expand internationally to satisfy consumer demand for top quality meat.

Currently, only 20% of the IB pigs slaughtered in Spain are produced under traditional systems [12] while a high proportion are reared indoors, crossed with Duroc sire lines, fed concentrate-based diets, and slaughtered at 140–150 kg BW [13]. The use of non-conventional raw materials may provide greater flexibility in the formulation of their diets, especially when these pigs are reared under intensive growing systems. 

A previous study reported that 100 g/kg of glycerin can partially replace wheat without affecting feed efficiency or nutrient digestibility in Iberian crossbred pigs from 50 to 100 kg BW [15], but the effects of adding glycerin in the late fattening stage of these pigs are still not known. Therefore, the objective of this research was to study the effects of supplementing the diet with crude glycerin on growth performance, nutrient digestibility, and blood parameters in finishing Iberian crossbred pigs reared indoors. 

## 2. Materials and Methods

All experimental procedures were approved by the University of Murcia Research and Ethics Committee (nº A-13170805) and followed the European Union guidelines for the care and use of research animals (Directive 2010/63/EU of the EU Parliament and the Council of 22 September 2010 on the protection of animals used for scientific purposes).

### 2.1. Experimental Design, Animals and Diets

The trial was conducted at a commercial fattening farm located in southeast Spain over a 74-days period. The study involved 192 Iberian × Duroc crossbred pigs sorted by initial body weight (average initial BW 96.0 ± 11.3 kg) and randomly allotted to pens balanced by gender (half barrows and half gilts). The pens were assigned to one of the three dietary treatments, with four pens per treatment. Each pen housed 16 animals (2 m^2^/pig) and it was provided with two feeders and two nipple drinkers in accordance with current Spanish legislation on minimum standards for the protection of pigs [16]. All pens were located in the same barn unit, and room temperature was kept between 18 and 26 °C. All animals were reared under the same environmental conditions under an intensive production regime. From the growing period to the beginning of the experimental fattening time (25–100 kg BW), animals were managed in accordance with standard commercial procedures. They received the same standard grower diets according to the Spanish Foundation for the Development of Animal Nutrition (Fundación Española para el Desarrollo de la Nutrición Animal (FEDNA) [17]. Pigs during experimental period received either a barley–wheat meal basal diet without glycerin (G0, control) or the basal diet with partial substitution of wheat with 5% (G5) or 10% (G10) crude glycerin. Diets were formulated to be isoenergetic and isoaminocidic, according to ideal protein concept, and to meet the recommendations for growing and finishing Iberian crossbred pigs [17] (net energy (NE) 9800 kJ/kg and ileal digestible standardized lysine (Lys), 4.6 g/kg). All feeds were manufactured using the same batch of ingredients. Ingredients and nutrient compositions of the diets are shown in Table 1. The crude glycerin used was obtained from a biodiesel production facility (Abengoa Bioenergía San Roque, Cadiz, Spain) that employs vegetable oils as feedstock. The crude glycerin had a content of 87.42% total glycerol, 7.98% moisture, 5.87% ash, 2.01% Na, 3.06% Cl, 0.05% K, 0.04% Ca, 0.24% P, and 0.05% methanol, and it was assumed a net energy of 12,057 kJ/kg in according to FEDNA [18]. Diets were presented in mash form and titanium dioxide (TiO_2_) at 5 g/kg (as-fed basis) was added as an indigestible marker to measure the apparent total tract digestibility (ATTD). Pigs were provided with feed and water ad libitum throughout the study.

### 2.2. Sample Collection and Analyses

All animals were weighed individually at the beginning and the end of the experimental period (days 0 and 74). The individual BW of pigs within each pen was used to calculate average daily gain (ADG) on an individual basis. Average daily feed intake (ADFI) per pen was recorded by weighing feed added to the feeders and feed remaining at the end of the experiment. The feed was administered in sacks, so it was controlled to avoid excessive wastage. Additionally, the feed conversion ratio (FCR) was calculated as the ratio between ADFI and ADG.

On day 49, blood samples were collected from four pigs from each pen (two barrows and two gilts) to determine serum concentrations of total protein, albumin, glucose, insulin, and IGF-1. Pigs within each pen were chosen according to their BW as being close to the average weight of the pen. Blood collection was performed via venipuncture of the jugular vein into 4.5 mL vacuum tubes without additives (Vacuette^®^, Greiner Bio-One, Kremsmünster, Austria). The samples were centrifuged for 10 min at 2500× *g* and the serum were stored at −80 °C until analysis.

On days 56 and 57, fecal samples were collected by stimulating-massaging the anus of two barrows and two gilts from each pen to calculate the ATTD of nutrients. The feces were pooled by pen and frozen at −20 °C for further analysis.

Feed samples were taken from each production batches. The dry matter (DM) content of the diets was determined by drying a sample in a convection oven at 105 °C for 8 h (AOAC 934.01 method, [19]). Fecal samples were dried at 60 °C until they reached a constant weight. Feed and fecal samples were ground to pass through a 1 mm mesh in an ultra-centrifugal mill (Retsch ZM 200; Retsch GmbH, Haan, Germany). Crude protein content was obtained through the Kjeldahl method (AOAC 984.13 A-D method) [18] and expressed as total N × 6.25. Diets were also analyzed for crude fiber (AOAC 920.39 method). The starch content was measured polarimetrically using the official analytical method [20]. The mineral content of diets and crude glycerin was determined by dry ashing using a muffle furnace at 550 °C for 4 h (AOAC 942.05 method), [18]). Ash was solubilized with 50 mL of 0.6 N nitric acid and subsequently filtered. The P content was determined by the official vanadate–molybdate method described in the Boletin Oficial del Estado [21]. The Ca content was detected by atomic absorption spectroscopy (Solaar M Series; Unicam, Cambridge, UK).

The moisture content of crude glycerin was determined by the Karl–Fischer method (AOAC 2001.12 method, [19]). The total glycerol and methanol contents of glycerin were analyzed by gas chromatography (TRACE GC Ultra, Thermo Electron Corporation, Rodano, Italy), using a 30 m × 0.25 mm × 0.25 μm capillary column (Tracsil TR-FFAP; Teknokroma, Barcelona, Spain) equipped with a flame ionization detector, as described by [4]. Titanium dioxide content in the diets and fecal samples was measured according to the method described by [22]. 

The apparent total tract digestibility of DM, organic matter (OM), and crude protein (CP) was calculated by using the equation:

ATTD (%) = (1 − ((nutrient in feces (g/kg DM) ÷ nutrient in diet (g/kg DM)) × (TiO_2_ in diet (g/kg DM) ÷ TiO_2_ in feces (g/kg DM)))) × 100 (1)

The serum samples were processed in the Interdisciplinary Laboratory of Clinical Analysis of the University of Murcia (Interlab-UMU, Spain). Serum total protein, glucose, and albumin were analyzed using an automatic analyzer (Olympus AU400; Olympus, Tokyo, Japan). Insulin was determined by an enzyme-linked immuno sorbent assay (ELISA) kit (Porcine Insulin ELISA, ref. 10-1200-01, Mercodia, Winston Salem, NC, USA). Insulin-like growth factor 1 (IGF-1) was determined with an automated chemiluminescent immunoassay (Immulite System, Siemens Health Diagnostics, Deerfield, IL, USA).

### 2.3. Statistical Analysis

Data of growth performance, coefficients of ATTD, and serum metabolites were analyzed using a general linear model in IBM SPSS Statistics software (IBM Corporation, Armonk, NY, USA). The model included the level of incorporation of glycerin (0%, 5%, and 10%) as the main effect for ADFI, FCR, and ATTD; and the level of incorporation of glycerin (0%, 5%, and 10%) and sex as the main effects and the first order interaction for BW, ADG, and blood parameters. The Shapiro–Wilks test was first used to assess whether serum data were normally distributed, and if not, the data were analyzed on a logarithmic scale. When the model was significant, differences between least squared means were investigated with Tukey’s test. The experimental unit was the pen for ADFI, FCR, and digestibility assay, while individual animals were taken as the experimental unit for BW, ADG, and serum parameters. The significance level was set at *p* ≤ 0.05 and trends at 0.10 ≥ *p* > 0.05.

## 3. Results

### 3.1. Growth Performance and Nutrient Digestibility

The effects of diet on growth performance are described in Table 2 and Table 3. No differences for initial and final BW were found (*p* > 0.05) in pigs on all diets. The ADG, ADFI, and FCR were not affected by dietary treatment.

The effects of glycerin addition on ATTD are shown in Table 4. Dietary glycerin did not affect the ATTD of DM, OM, and CP.

### 3.2. Blood Parameters

**Figure 1:**
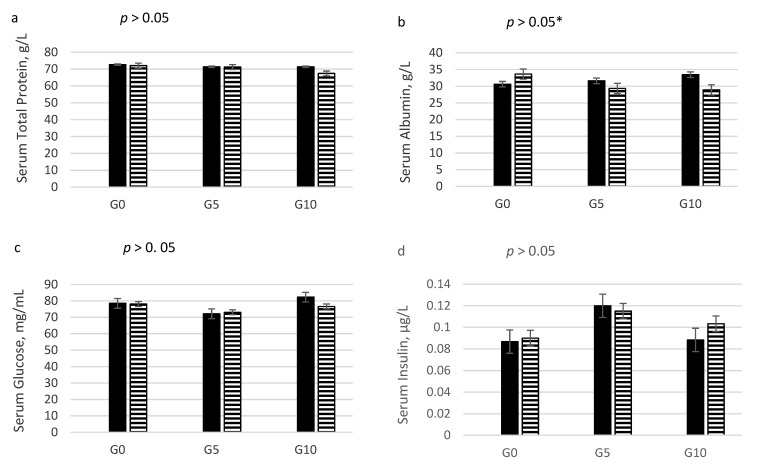

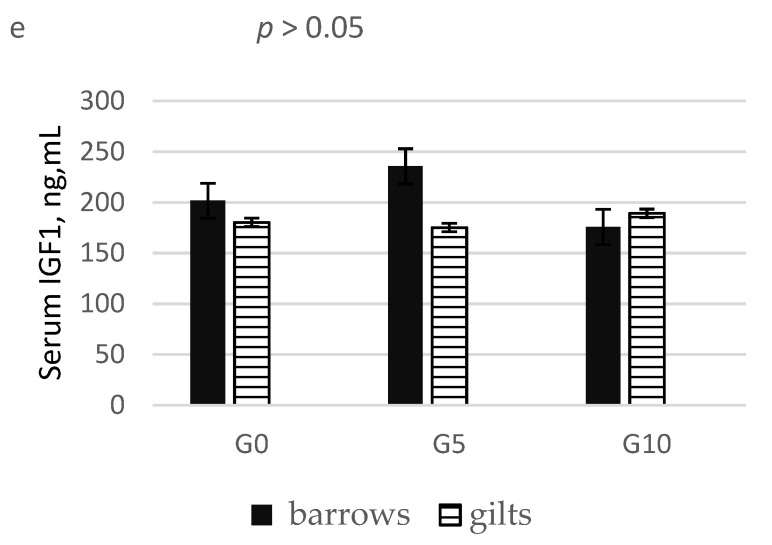
Effects of adding glycerin to feed on serum (**a**) total protein, (**b**) albumin, (**c**) glucose, (**d**) insulin, (**e**) IGF1. G0: diet with 0% crude glycerin. G5: diet with 5% crude glycerin. G10: diet with 10% crude glycerin. Data show least squared means ± standard error. *n*= 16 animals per dietary treatments. * *p* > 0.05, except for albumin diet × sex *p*-value = 0.036.

Serum concentrations of total protein, albumin, insulin, and IGF-1 were not affected by dietary treatment (Figure 1). A trend of lower glucose levels was found in G5 compared to G10 (*p* = 0.094). A significant interaction between diet and sex was observed for albumin concentration.

## 4. Discussion

Crude glycerin has been used as an energy source to replace cereals in animal diets, thus increasing the supply of raw materials while reducing production costs [23,24,25]. Numerous studies have been performed using glycerin in diets at different stages of pig production: in sows [5,26], weaning pigs [2,9,27], growing and finishing pigs [4,28]. All of them were carried out with conventional pig genotypes. However, Iberian pigs represent 11% of the pig herds in Spain [29]. The breeding of IB is different, with a high potential for fat accumulation and lower performance compared with white pig genotypes [30]. As intensive rearing of IB pigs is becoming more common, the search for alternative ingredients that do not compete with human food and are locally available is essential for the sustainability of this type of production.

In this research, we used Iberian crossbred pigs in the late fattening stage and found no effects of incorporating crude glycerin up 10% on performance traits. Similarly, Orengo et al. [15] showed that 100 g/kg of glycerin could partially replace wheat without affecting feed efficiency in IB pigs up to 100 kg BW. Other researchers showed no effects or positive effects on growth performance by incorporating glycerin in commercial finishing pig diets at 5% [31], 8% [28], 15% [9], or 18% [32]. The origin and quality of glycerin, even the cereal replaced, in the above studies were not all the same, and this could partly explain the different results.

In a study on pigs with similar body weight to ours, Della Casa et al. [33] using Italian Duroc × Italian Large White pigs up to 160 kg BW, observed no effects of replacing corn with 5% glycerol in the diet, whereas pigs fed 10% glycerol showed reduced growth and a poorer feed–gain ratio. They attributed their results to free glycerol excretion via the urine, and they suggested that the higher the glycerol content of the diet, the greater the elimination due to liver glycerol kinase saturation. In this sense, Papadomichelakis et al. [34] concluded that pigs need to adapt to the inclusion of crude glycerin in their diet, and the efficiency of glycerol kinase may be related to the duration of crude glycerin consumption. They found that post-weaned pigs, after adapting to increasing levels of crude glycerin, efficiently absorbed and metabolized up to 150 g/kg diet as a dietary energy source.

The IB pig is a rustic breed that has not undergone intense genetic selection. It is characterized by its high appetite, fatness, and protein turnover ratio, which result in low lean growth efficiency, but high fat deposition compared with other breeds [30]. IB pigs have been suggested to have more active lipogenesis than other breeds [35]. Torres-Rovira et al. [36] assumed that when there is a positive energy balance, the Iberian sow’s fat deposition is first intramuscular throughout the carcass, then it is also subcutaneous and visceral. In growing animals, it has been shown that adipocyte hypertrophy is the most important issue affecting intramuscular fat content [30]. Hypertrophy is driven by triglyceride accumulation in mature adipocytes. In this sense, glycerin may contribute to the deposition of triglycerides in adipose tissue in these fatty breeds, which would make them more tolerant to higher amount of glycerin than lean breeds. Glycerol kinase saturation in these fatty breeds may be attained at higher levels than lean breeds. Although future studies are required to confirm it.

Glycerin is considered an energetic ingredient that is easily digested and metabolized by pigs [37]. In our case, incorporating up to 100 g/kg crude glycerin, with a similar energy value to wheat, achieved similar nutrient digestibility to the control. Morales et al. [38] showed that IB pigs have higher feed intake, increased stomach content, lower digesta content in the hindgut compartment, and a shorter time of digesta retention for fermentation than conventional pigs, and this leads to a lower digestibility coefficient of some nutrients. Most digestibility studies of conventional crossbred pigs conclude that the incorporation of crude glycerin does not affect nutrient digestibility in growing–finishing pigs [4,9,27]. Orengo et al. [15] showed no significant effect of incorporating of 10% glycerin in diets for IB crossbred pigs up to 100 kg on nutrient digestibility, as we observed for the last fattening phase in the current study.

Crude glycerin is not a standardized product, and its digestibility depends on its glycerol content [37]. Early studies used glycerin with at least 85% glycerol, so it was of good quality, and this is probably one of the reasons why an effect on digestibility was not found.

In pigs, dietary glycerol is absorbed by the stomach and intestines and transported to the liver to be metabolized. Depending on the nutritional status of the animal, glycerol can be used in lipid formation or energy production. Glycerol kinase is the liver enzyme that converts glycerol to glycerol 3-phosphate, which in turn is converted to glucose via gluconeogenesis or oxidized for energy via glycolysis or the citric acid cycle [39]. For this reason, incorporating glycerol in feed could alter some parameters related to metabolism. However, this hypothesis is not confirmed in our study, as we found no significant effect of glycerin in the diet on serum parameters, even at the highest levels (up to 10%).

These results are in line with numerous studies that incorporated glycerin at different levels in the diets of growing–finishing lean-type pigs: 18% [27], 16% [11], 14% [10], 10% [7], and 5% [4], and of IB crossbred pigs up to 100 kg [15], with no observed effect on blood parameters (total protein, albumin, glucose, insulin, and IGF-1) similar to our results. Thus, according to Mazzo et al. [40] we can suggest that the use of components with a high glycemic index such as glycerin does not necessarily lead to changes in energy metabolism parameters.

However, other parameters related to lipid metabolism, such as triglycerides and cholesterol, which were not analyzed in this study, are more frequently affected by glycerin addition [10,23,39,41,42]. In this sense, Narayan and Mcmullen [43] showed that glycerin metabolism in the livers of mice and birds after chronic intake has a stimulatory effect on the synthesis of free fatty acids, triglycerides, cholesterol, chylomicrons, and plasmatic lipoproteins.

The IB breed has greater potential for fat accumulation and more active lipid and glucose metabolism than other breeds [12,30,44]. Furthermore, differences in hepatic metabolism between IB and conventional breeds has been described [45]. Hepatocytes from IB pigs had greater glucose production from gluconeogenic precursors, increased albumin synthesis and lower hepatic IGF-1 synthesis. In this study, serum parameters were not affected by glycerin addition, but we observed an interaction between diet and sex for albumin concentration. A possible explanation for our results could be that males have greater backfat thickness and fat content than intact females [6,46]. This may condition the behavior of pigs fed glycerin diet since albumin is a liver protein related to the capacity for fatty acid transport from blood to adipocytes [44].

## 5. Conclusions

Our results show good acceptance of dietary glycerin by Iberian pigs, which may tolerate better than lean breeds. The inclusion of crude glycerin up to 10% in feeds of Iberian crossbred pigs during the 100 to 145 kg phase had similar growth performance, feed digestibility, or serum metabolites as total proteins, albumin, glucose, insulin, and IGF1 to control diet, so glycerin could be considered an energetic ingredient to partially replace wheat in the last stages of IB fattening. 

## Figures and Tables

**Table 1:** Ingredients and composition of diets (as fed-basis).

Item	Diet ^1^		
	G0	G5	G10
Ingredient, %			
Barley	45.00	45.00	45.00
Wheat	34.52	27.45	20.55
Wheat bran	11.35	11.55	11.50
Soybean meal (47% crude protein)	6.11	7.64	9.19
Crude glycerin	-	5.00	10.00
Calcium carbonate	1.13	1.43	1.78
Animal fat	0.50	0.50	0.50
Titanium dioxide	0.50	0.50	0.50
Salt	0.35	0.30	0.25
Monocalcium phosphate	0.32	0.32	0.33
Vitamin-trace mineral premix ^2^	0.30	0.30	0.30
L-Lys 50 (50% Lys)	0.02	-	-
Calculated composition ^3^			
NE, kJ/kg	9800	9800	9800
CP, %	13.00	13.00	13.00
Ca, %	0.65	0.76	0.90
P total, %	0.47	0.47	0.46
Ether extract, %	2.53	2.44	2.35
C18:2 Linoleic acid	0.85	0.81	0.78
Starch	47.40	43.30	39.20
Cellulose	4.14	4.08	4.00
Standardized ileal digestible Lys, %	0.46	0.46	0.46
Analyzed composition, % on dry matter (DM)basis except for DM			
DM	90.70	91.10	89.70
CP	14.22	13.39	13.71
Crude fiber	4.08	3.70	3.58
Ether extract	2.75	2.66	2.60
Starch	55.00	51.10	45.50
Ash	6.07	6.63	7.54
Ca	0.89	1.16	1.56
P total	0.55	0.70	0.73
Glycerol	0.00	5.59	8.59
EN, kJ/kg ^4^	10,970	10,867	10,702

^1^ Dietary treatments: 0, 5 and 10% crude glycerin included in G0, G5, and G10, respectively. ^2^ Provided (per kg of complete diet): 7000 IU vitamin A; 1500 IU vitamin D_3_, 20 mg vitamin E, 4 mg vitamin B_2_, 1.5 mg vitamin B_6,_ 0.020 mg vitamin B_12_, 20 mg of niacin; 8 mg of calcium pantothenate; 100 mg of choline chloride; 75 mg of zinc oxide; 40 mg of manganese (II) oxide, 75 mg ferrous sulfate heptahydrate, 12 mg cupric sulfate pentahydrate, 0.15 mg sodium selenite, 1 mg potassium iodate, 0.1 mg basic cobaltous carbonate monohydrate, 500 FTU phytase (EC 3.1.3.8,(NeutralSCA, Cargill, Madrid, España). ^3^ According to FEDNA [18]. ^4^ Calculated from the Evapig^®^ prediction equations. Energy value of glycerol was assumed as an available carbohydrate.

**Table 2:** Effects of sex and glycerin addition to feed on body weight and average daily gain of Iberian crossbreed pigs.

Item	Diet ^1^			Sex		SEM ^2^	*p*-Value		
	G0	G5	G10	Barrows	Gilts		Diet	Sex	D × S
N	64	64	64	96	96				
Initial BW, kg	95.48	96.52	95.48	98.8	91.8	1.566	0.809	0.452	0.473
Final BW, kg	137.6	142.8	138.0	141.3	137.6	2.196	0.627	0.765	0.332
ADG, kg	0.57	0.62	0.60	0.57	0.62	0.010	0.152	0.458	0.151

^1^ Dietary treatments: 0, 5 or 10% crude glycerin included in G0, G5, and G10, respectively. ^2^ SEM, standard error of the mean. BW, body weight; ADG, average daily gain. D × S, glycerin × sex.

**Table 3:** Effects of glycerin addition to feed on average daily feed intake and feed conversion ratio of Iberian crossbred pigs.

Item	Diet ^1^			*p*-Value	
	G0	G5	G10	SEM ^2^	Diet
N	4	4	4		
ADFI, kg	2.67	2.78	2.76	0.047	0.618
FCR, kg:kg	4.69	4.45	4.65	0.058	0.345

^1^ Dietary treatments: 0, 5, and 10% crude glycerin included in G0, G5, and G10, respectively. ^2^ SEM, standard error of the mean. ADFI, average daily feed intake; FCR, feed conversion ratio (kg feed:kg body weight).

**Table 4:** Effects of glycerin addition to feed on apparent total tract digestibility of nutrients in Iberian crossbred pigs.

Item	Diet ^1^			SEM	*p*-Value
	G0	G5	G10		Diet
Replicates	4	4	4		
DM ^2^	78.6	79.3	77.1	0.421	0.141
OM	81.6	82.3	81.4	0.365	0.571
CP	72.6	75.2	68.3	1.252	0.118

^1^ Dietary treatments: 0, 5, and 10% crude glycerin included in G0, G5, and G10, respectively. ^2^ Percentage of apparent total tract digestibility of dietary components. DM, dry matter; OM, organic matter; CP, crude protein. SEM, standard error of the mean.

## Data Availability

Data sharing is not applicable to this article.
